# Cost-effectiveness of endovenous laser ablation of the great saphenous vein in patients with uncomplicated primary varicosis

**DOI:** 10.1186/s12872-015-0130-1

**Published:** 2015-10-28

**Authors:** Thomas Luebke, Jan Brunkwall

**Affiliations:** Department of Vascular and Endovascular Surgery, University Hospital of Cologne, Kerpener Str. 62, 50937 Cologne, Germay

## Abstract

**Background:**

Although widely applied, the cost-effectiveness of endovenous laser ablation (EVLT) for varicose veins has not been established.

**Methods:**

Cost-effectiveness analysis was performed on the evaluation of EVLT for the treatment of uncomplicated varicose veins by using published data from randomizd clinical trials regarding the costs and the quality of life. Incremental cost per quality-adjusted life year (QALY) gained at 6 months following treatment was calculated. Sensitivity analysis was carried out to investigate the uncertainty associated with the results of our analysis.

**Results:**

Over the time horizon of 1–6 months, it was found that the incremental cost of EVLT compared with conventional surgery was €466.66 and the incremental effect was −0.007 QALY at 1 month, −0.0075 QALY at 3 months and 0.0 QALY at 6 months. This shows that the strategy “EVLT” was dominated by the strategy “HL/S” at any time point for the base cases analyses. The results of various alternative economic evaluations indicated that EVLT may be a potentially cost effective (i.e. incremental cost effectiveness ratio of between €12158.67 and €514721.67 per QALY, respectively) treatment option compared to conventional surgical treatment for varicose veins with a certainty between 54.9 and 98.8 %.

**Conclusion:**

For patients with uncomplicated varicose veins and evidence of saphenofemoral reflux, surgical treatment for varicose veins offers a robust health benefit for relatively less costs compared to EVLT.

## Background

Varicose veins are a common problem of Western adults. The Framingham Study (USA) demonstrated a biannual incidence rate of varicose veins of 2.6 % in women and 2.0 % in men. The prevalence of varicose veins in Western populations has been estimated to be about 25–30 % among women and 10–20 % in men [1]. Therefore, they represent a huge burden on the health systems. Besides the procedural workload, patients with varicose veins account for large numbers of outpatient attendances in primary and secondary care [2].

Although varicose veins may be asymptomatic, especially in the early periods of the disease, frequent symptoms include localised swelling, heaviness, cramps and aches, chronic localised fatigue, itching and tingling. More serious symptoms as an indicator for chronic venous insufficiency, eg superficial thrombophlebitis, bleeding, lipodermatosclerosis with eczema and skin hyperpigmentation may occur in a certain proportin of these patients as a prelude to venous ulceration [1, 3]. As a result, varicose veins affect patients’ quality of life (QoL) negatively [4–7].

Over the past decades, the standard surgical treatment of the insufficient great saphenous vein (GSV) has been high ligation and stripping (HL/S) combined with phlebectomies [8]. The results of this procedure are long lasting and HL/S has been shown to improve disease-specific and general quality of life of the patients with primary varicosis [6, 9]. However, HL/S is often performed as a day-case or inpatient operation with general or regional anesthesia, which increases costs, although it may also be performed with tumescent anesthesia with good patient comfort [10]. Furthermore, HL/S is oftenly associated with a period of recuperation and time off work and the possibility of complications [2] like postoperative pain and bruising, bleeding, groin infection, phlebitis, and nerve damage [11, 12]. Recurrence rates ranging from 20 to 80 % have been reported between 5 and 20 years after surgery [13].

Recently, minimally invasive alternatives have been developed for the treatment of varicose veins, such as endovenous laser ablation of the GSV (EVLT), which may be performed in an outpatient setting with the patient receiving local anaesthesia or light sedation. This therapy has been shown to have similar short-term results for up to 3 to 5 years concerning complete occlusion of the GSV and freedom of reflux, compared to HL/S [14, 15]. Furthermore, in comparison to HL/S, EVLT has been reported to be associated with comparable complication rates, but is characterized by higher patient preference, reduced postoperative pain, shorter sick leave, a faster resumption of the normal activities and a faster return to work [14–16]. Such advantages may compensate the extra costs of the laser equipment, which include a generator and disposable introducer catheters and fibers [17, 18].

Despite the popularity and strong evidence demonstrating the clinical effectiveness of EVLT, only few formal cost-effectiveness analysis of this alternative techniques have been previously performed. This is important because unless the increased clinical effectiveness of an intervention justifies its incremental cost, policymakers cannot justify allocating resources for its widespread adoption.

Consequently, the specific aims of this study are as follows: (1) to investigate health-related quality of life (HRQoL) after EVLT and conventional surgery of the great saphenous vein for the treatment of varicose veins; (2) to compare the cost-effectiveness of conventional surgery and EVLT; and (3) to investigate and quantify the uncertainty associated with the results of our analysis.

## Methods

To investigate the cost-effectiveness of EVLT, we constructed a decision-analytic model. For the present analysis, costs were transformed and reported in Euros. To minimise bias, it was intended that costs and health benefits associated with EVLT and surgery should be sourced from randomized controlled trials (RCT) only. Therefore, a multiple electronic health database search including Medline, Embase, Ovid, Cochrane Database of Systematic Reviews, and Cochrane Database of Abstracts of Reviews of Effectiveness (DARE), was performed to identify RCTs examining the costs of EVLT as a part of the trial, compared to HL/S. The literature search yielded two RCTs regarding the direct and total costs. In the study by Rasmussen [17], calculations of costs were based on the standard fee for HL/S with the addition of the costs of EVLT equipment and the standard salary and productivity level in Denmark. The impact of sick leave on costs was corrected for weekends. From the second RCT (REACTIVE trial) [2] additional economic data on surgery for varicose veins were obtained. In the REACTIVE trial, NHS treatment costs included all NHS contacts with primary and secondary healthcare services and treatments and medications administered. Unit costs for all resources used by trial patients were obtained for the financial year 2002–3 and were obtained using national sources wherever possible, including the Personal Social Services Research Unit Database [19], NHS Reference Costs [20], and the BNF [21]. Where national costs were unavailable, local unit costs were obtained from the finance departments at each of the two participating hospitals. The impact of the time to resume work on total costs for HL/S and EVLT was extracted from 4 RCTs [2, 17, 22, 23].

From four RCTs [17, 22–24] data on quality of life (QoL) were obtained for EVLT using the medical outcomes Short Form 36 (SF-36) health survey. The SF-36 is a widely used generic QoL instrument that has been demonstrated to be valid, reliable, and sensitive [5–7, 25]. It consists of 36 individual items aggregated to form eight domains: Physical Functioning (PF), Role-Physical (RP), Bodily Pain (BP), General Health (GH), Vitality (VT), Social Functioning (SF), Role Emotional (RE), and Mental Health (MH) [26]. Each domain is scored from 0 (worst score) to 100 (best score) [24].

From three RCTs [2, 17, 24] SF-36 data for surgery were collected. The REACTIVE trial [2] provided Visual Analogue Scale (VAS) and Standard Gamble (SG) data on surgery, and Michaels et al. [27] provided VAS data on surgery, as well. The visual analogue scale, and standard gamble techniques are direct measures that provide information regarding the health status.

Neither study directly assessed utility of the treatment options. A method of imputing HUI2-II utility scores from SF-36 scores has recently become available [28], even without having individual patient data using the algorithm by Nichol et al. [28]. SG utilities for the patients’ VAS score were derived using a transformation function to convert adjusted VAS values to SG utility scores. VAS scores were first transformed from a 0–100 scale to a 0.00–1.00 scale. Then, power functions were used to transform the data to SG utility scores. Power conversion is the most common transformation function used for mapping the relationship between VAS scores and SG utilities [29, 30]. In the present analysis, one function, previously described by Torrance [30], 1982 was used to perform the transformations [31]. Finally, Kovacs and colleagues [32] recently published a survey where they have examined the relationship between VAS pain and utility (as assessed by the EQ-5D) in patients with low back pain. Using regression methods they found that a 1 mm increase in VAS (on a 0–100 scale) is associated with a −0.035 decrement in utility.

By transforming QoL data into utility scores for both treatments, there was no bias in our analysis against or in favour the one or the other treatment option. The calculated reductions in utility after EVLT and surgery are shown in Table 1.

**Table 1:** Summary of utility parameters used in the decision analytic model

	EVLT QALY	Surgery QALY	Time point	Data source for calculation, distribution
Base case 1 (+ Alternative 4, 7)	SF-36 derived HUI2-II score: 1.360; QALY: 0.1133	SF-36 derived HUI2-II score: 1.430; QALY: 0.1192	1 month	Rasmussen [1], triangular
Base case 2 (+ Alternative 5, 8)	SF-36 derived HUI2-II score:1.440; QALY: 0.3600	SF-36 derived HUI2-II score:1.470; QALY: 0.3675	3 months	Rasmussen [1], triangular
Base case 3(+ Alternative 6, 9)	SF-36 derived HUI2-II score:1.470; QALY: 0.735	SF-36 derived HUI2-II score:1.470; QALY: 0.735	6 months	Rasmussen [1], triangular
Alternative 10, 12, 13	SF-36 derived HUI2-II score: 1.480; QALY: 0.1709	SF-36 derived HUI2-II score: 1.390; QALY: 0.1605	6 weeks	Mekako [24], triangular
Alternative 11, 15, 14	SF-36 derived HUI2-II score: 1.520; QALY: 0.3510	SF-36 derived HUI2-II score:1.420; QALY: 0.3279	12 weeks	Mekako [24], triangular
Alternative 16, 17	SF-36 derived HUI2-II score: 1.467; QALY: 0.735	SF-36 derived HUI2-II score: 1.473; QALY: 0.735	6 months	Rasmussen [1], triangular
Alternative 18, 19	VAS disutility as to Kovacs: EVLT1: −0.28 EVLT2: −0.175; QALY: −0.0053846/−0.0033654	VAS disutility as to Kovacs: −0.28; QALY: −0.0053846	Day 7	Darwood [23], triangular
Alternative 20, 21	VAS disutility as to Kovacs: EVLT1: −0.385 EVLT2: −0.630; QALY: −0.0074038/−0.0121154	VAS disutility as to Kovacs: −0.49; QALY: −0.0094231	Mean day 1–7	Darwood [23], triangular
Alternative 22, 24	VAS disutility as to Kovacs: −0.606; QALY: −0.0126923	VAS disutility as to Kovacs: −0.602; QALY: −0.0115769	Day 7	Kalteis [22], triangular
Alternative 23, 25	VAS disutility as to Kovacs: −0.039; QALY: −0.00325	VAS disutility as to Kovacs: −0.087; QALY: −0.00715	Day 28	Kalteis [22], triangular

Analysis of outcomes was on an intention-to-treat (ITT) basis. The effects of the interventions on QoL were measured in quality-adjusted life years (QALYs). Cost-effectiveness ratios are reported in Euros per QALY. Because most capital expenditure and effects occurred within 6 months of the primary procedure in our analysis, cost and effects were not discounted.

Adverse events were not included in the present analysis because according to a multidisciplinary Guideline Development Group, which currently develops the NICE guidelines for the diagnosis and management of varicose veins, adverse event of the different interventions are similar to the extent that they can be neglected in health economic models [33]. It was therefore assumed that any disutility (and costs) associated with short-term complications was equivalent between EVLT and stripping.

The primary analysis reflected a comparison of costs and QALYs measured using the SF-36- and VAS- derived utilities (HUI2-II, SG) from 4 weeks up to 6 months. Incremental cost-effectiveness ratios (ICERs) were estimated for each group. It was assumed that the probability of survival was equivalent for EVLT and HL/S. Therefore, the incremental cost per QALY of EVLT compared to conventional surgical approaches was driven by differences in healthcare costs and utility (quality of life) gain.

To calculate the incremental QALY associated with EVLT and surgery, we assumed that recovery were constant after both techniques between discharge and 4 weeks. Finally, we did not account for differences in utility before discharge when calculating QALYs. In the case where only one cost or utility value was available from the literature, a range was imputed, and sensitivity analyses were conducted based on assumed standard deviations of the point estimate.

One-way sensitivity analyses and alternative analyses were conducted to test the robustness of the results to changes from the base case, by using the various HUI2-II utilities and VAS derived SG utilities as the measure of health outcome and applying the different total costs (caused by the different times to resume work among the trials) for treatment of varicose veins reported in the RCTs included. Probabilistic analysis was performed by using two-dimensional Monte Carlo simulation, with 10.000 model recalculations. All values from the clinical trials analyzed were recorded, and the mean and standard deviation values were calculated. The maximum and minimum values associated with the costs and utilities were calculated by doubling the difference between the mean and upper and lower quartiles obtained from reported costs and utilities in the RCTs. In instance where only one point estimate for costs or utilities were available, the standard deviation was assumed to be equal to 0.5 times the mean point estimate value. Alternative analysis was also performed to further investigate the uncertainty associated with our estimates of QALY payoffs using different combinations between the utilities and the costs.

**Fig. 1:**
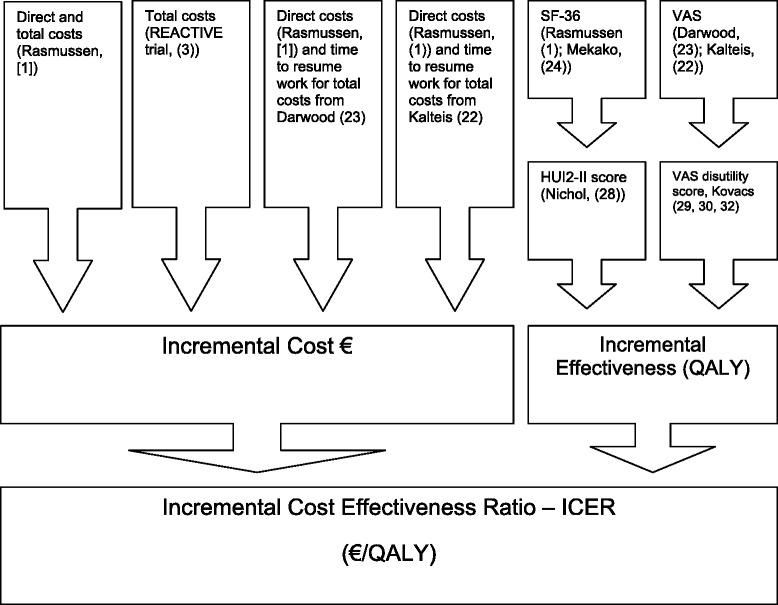
Summary of the decision analytic model used and the sources of model cost and utility parameters

Our analysis is summarized in Fig. 1. A full list of all model parameters is given in Tables 1 and 2.

**Table 2:** Summary of cost parameters used in the decision analytic model

	Treatment	Time point	Unit costs €	Source	Details
Base case 1–3 (+ Alternative 10, 11, 16, 18, 20, 22, 23)	EVLT	1, 3, 6 months, day 7, 28	1390.66	Rasmussen [1]	Direct costs
Base case 1–3 (+ Alternative 10, 11, 16, 18, 20, 22, 23)	Surgery	1, 3, 6 months, day 7, 28	924.00	Rasmussen [1]	Direct costs
Alternative 4–6 (+ Alternative 12, 14, 17)	EVLT	1, 3, 6 months	3396.40	Rasmussen [1]	Total costs, time to resume work 7.0 ± 6.0 (1–31) days
Alternative 4–6 (+ Alternative 12, 14, 17)	Surgery	1, 3, 6 months	3084.50	Rasmussen [1]	Total costs, time to resume work 7.6 ± 4.9 (1–28) days
Alternative 7–9 (+ Alternative 13, 15)	EVLT	1, 3, 6 months	3396.40	Reactive (2)	Total costs, time to resume work 7.0 days
Alternative 7–9 (+ Alternative 13, 15)	Surgery	1, 3, 6 months	4458.00	Reactive [3]	Total costs, time to resume work 12.4 days
Alternative 19, 21	EVLT	-	2530.66	Darwood [23] - TRW / Rasmussen [1] – direct costs	Total costs, 4 days time to resume work, EVLT1: 12 W pulsed; EVLT2: 14 W continuous
Alternative 19, 21	Surgery	-	5769.00	Darwood [23] - TRW / Rasmussen [1] – direct costs	Total costs, 17 days time to resume work,
Alternative 24, 25	EVLT	-	7090.66	Kalteis [22] - TRW / Rasmussen [1] – direct costs	Total costs, 20 days to resume work
Alternative 24, 25	Surgery	-	4914.00	Kalteis [22] - TRW / Rasmussen [1] – direct costs	Total costs, 14 days to resume work

*TRW* time to return to work in days

Data from the assessments and questionnaires were coded and analysed using SPSS, Excel and DATA (a specialist decision modelling software package, TreeAge-Pro; TreeAge, Williamstown, Mass).

## Results

Table 2 shows the healthcare costs of EVLT, and HL/S with and without inclusion of various indirect costs associated with different time intervals to resume work.

### Costs

The majority of the additional direct costs of EVLT compared to HL/S was the cost of the laser equipment. As expected, the direct costs in the base case scenarios (months 1, 3, and 6) were much higher for the EVLT group compared to the surgically treated group (Table 2). In the alternative cases 4–6, 12, 14, and 17 (for sensitivity analysis), the mean time to resume work (TRW) (7.6 vs 7.0 calendar days) did not differ significantly between the HL/S and EVLT groups. Under these conditions, the mean cost of the HL/S procedure was €3084 ($3948 US) when loss of productivity was included compared with €3396 ($4347 US) in the EVLT group. Thus, the direct procedure-related costs were higher in the EVLT group, but the difference between the groups was somewhat reduced by the lower loss of productivity among the EVLT patients.

### HRQoL

Table 1 shows the SF-36 derived HUI2-II scores, and VAS-derived SG scores from various sources according to the treatment options for a time period up to 6 months. For the base case scenarios, the health status of the EVLT group was lower than that of the HL/S group one and 3 months after treatment. After 6 months, the EVLT-group and the surgical group had the same health status, regarding the base case analyses.

### Cost-effectiveness

Over the period of 1–6 months, it was found that the incremental cost of EVLT compared with conventional surgery was €438.89 and the incremental effect of EVLT was −0.007 QALY at 1 month, −0.0075 QALY at 3 months and 0.0 QALY at 6 months. Under these conditions, the strategy “EVLT” was dominated by the strategy “HL/S” at any time point, for the base cases analyses.

**Fig. 2:**
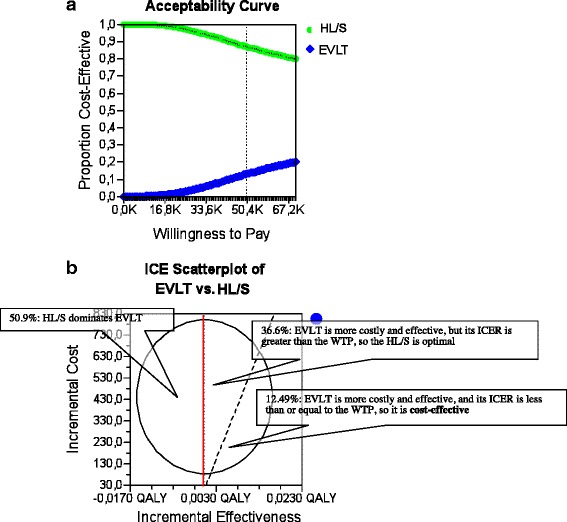
**a** Base case 1: Cost-effectiveness acceptability curve. The graph gives the probability that HL/S (87 %) or EVLT (13 %) would be considered cost effective for a €50000 threshold of willingness to pay. **b** Monte Carlo simulation of EVLT vs. HL/S A 10,000-iteration Monte Carlo simulation of a patient undergoing EVLT. The incremental cost and incremental effectiveness of EVLT compared with HL/S is plotted for each iteration. Of 10000.00 iterations, 5090.00 showed HL/S to be optimal by possessing ICERs below the €50,000/QALY threshold (northwest quadrant)

**Fig. 3:**
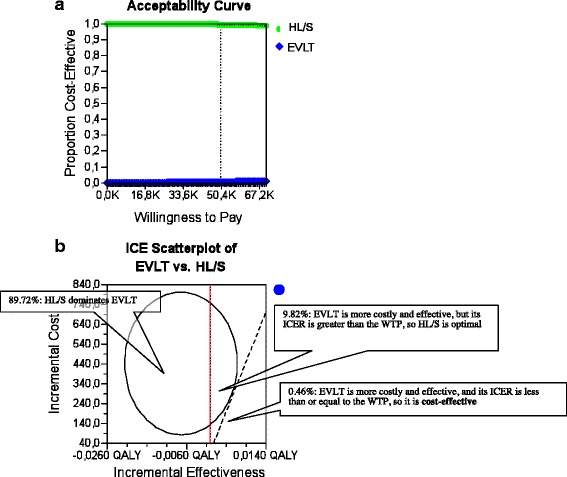
**a** Base case 2: Cost-effectiveness acceptability curve. The graph gives the probability that HL/S (100 %) or EVLT (1 %) would be considered cost effective for a €50000 threshold of willingness to pay **b** Monte Carlo simulation of EVLT vs. HL/S A 10,000-iteration Monte Carlo simulation of a patient undergoing EVLT. The incremental cost and incremental effectiveness of EVLT compared with HL/S is plotted for each iteration. Of 10000.00 iterations, 8972.00 showed HL/S to dominate EVLT by possessing ICERs below the €50,000/QALY threshold (northwest quadrant)

The 10000-iteration Monte Carlo simulation for the base case analysis after 1 month is shown in the acceptability curve ICER scatter plot in Fig. 2a, b, c. The ellipse encloses 95 % of the model recalculations; the dotted-diagonal line represents the cost-effectiveness threshold, plotted at € 50,000/QALY, which is commonly applied in the published literature. In 50.9 % of model recalculations, HL/S dominates EVLT (less costly and more effective), in 36.6 % of model recalculations, EVLT is more costly and effective, but its ICER is greater than the willingness to pay (WTP), so HL/S is optimal, and in 12.49 % of model recalculations, EVLT is more costly and effective, and its ICER is less than or equal to the WTP. Consequently, EVLT is cost-effective, with 12.49 % certainty at a threshold of € 50,000/QALY in our base case 1. For the base case analysis 2 after 3 months, the 10,000-iteration Monte Carlo simulation is shown in the acceptability curve and ICER scatter plot in Fig. 3a, b, c. In 89.72 % of model recalculations, HL/S dominates EVLT (less costly and more effective), in 36.6 % of model recalculations, EVLT is more costly and effective, but its ICER is greater than the WTP, so HL/S is optimal, and in 0.46 % of model recalculations, EVLT is more costly and effective, and its ICER is less than or equal to the WTP. Consequently, EVLT is cost-effective, with 0.46 % certainty at a threshold of € 50,000/QALY in our base case 2.>

### Sensitivity analysis

**Fig. 4:**
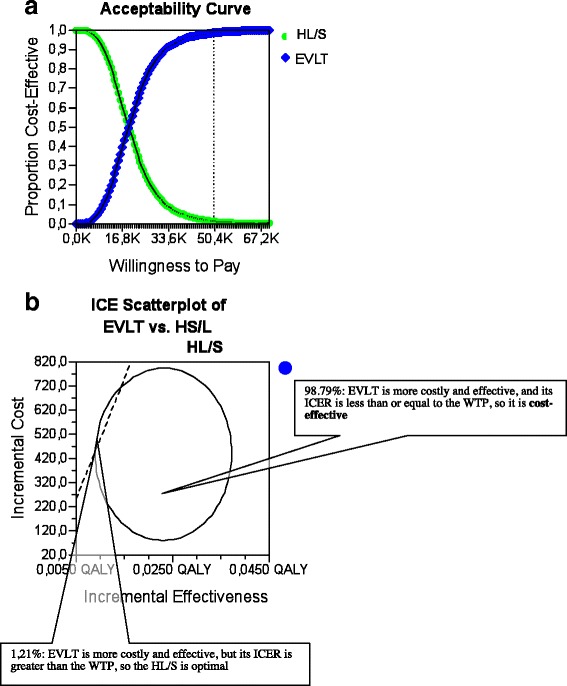
**a** Alternative analysis 11: Cost-effectiveness acceptability curve. The graph gives the probability that HL/S (1 %) or EVLT (99 %) would be considered cost effective for a €50000 threshold of willingness to pay. **b** Monte Carlo simulation of EVLT vs. HL/S. A 10,000-iteration Monte Carlo simulation of a patient undergoing EVLT. The incremental cost and incremental effectiveness of EVLT compared with HL/S is plotted for each iteration. Of 10000.00 iterations, 9879.00 showed EVLT to be cost-effective by possessing ICERs below the €50,000/QALY threshold

**Fig. 5:**
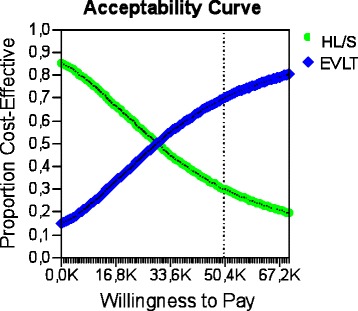
Alternative analysis 12: Cost-effectiveness acceptability curve. The graph gives the probability that HL/S (30%) or EVLT (70%) would be considered cost effective for a €50000 threshold of willingness to pay

A number of alternative analyses of cost effectiveness were carried out to test assumptions made in the base case analyses and to improve the generalizability of the results, including using HUI2-II scores at different time points, using the VAS-derived SG scores as the measure of health outcome, and using alternative total costs for EVLT and HL/S (Tables 3, 4, 5, 6, 7; Fig. 4a, b; Fig. 5).

**Table 3:** EVLT was dominated by HL/S (*n* = 8)

	QoL better for	TRW shorter for	Costs higher for	Time point
Alternative 4	HL/S	Equal	Total costs, EVLT	1 month
Alternative 5	HL/S	Equal	Total costs, EVLT	3 months
Alternative 6	Equal	Equal	Total costs, EVLT	6 months
Alternative 17	HL/S	Equal	Total costs, EVLT	6 months
Alternative 16	HL/S	-	Direct costs, EVLT	6 months
Alternative 18	EVLT	-	Direct costs, EVLT	Day 7
Alternative 22	HL/S	-	Direct costs, EVLT	Day 7
Alternative 24	HL/S	HL/S	Total costs, EVLT	Day 7

**Table 4:** HL/S was dominated by EVLT (*n* = 5)

	QoL better for	TRW shorter for	Costs higher for	Time point
Alternative 9	equal	EVLT	Total costs, HL/S	6 months
Alternative 13	EVLT	EVLT	Total costs, HL/S	6 weeks
Alternative 15	EVLT	EVLT	Total costs, HL/S	12 weeks
Alternative 19	Equal or EVLT (dependent on the W-impulse used)	EVLT	Total costs, HL/S	Day 7
Alternative 21	EVLT / HL/S (dependent on the W-impulse used)	EVLT	Total costs, HL/S	Day 1 –7

**Table 5:** No strategies were clearly dominated by any other (*n* = 8)

	QoL better for	TRW shorter for	Costs higher for	Time point	Σ
Alternative 7	HL/S	EVLT	Total costs, HL/S	1 month	EVLT: 83.9 % optimal
Alternative 8	HL/S	EVLT	Total costs, HL/S	3 months	EVLT: 89.9 % optimal
Alternative 10	EVLT	-	Direct costs, EVLT	6 weeks	EVLT: 58.9 % CE
Alternative 11	EVLT	-	Direct costs, EVLT	12 weeks	EVLT: 98.8 % CE
Alternative 23	EVLT	-	Direct costs, EVLT	Day 28	HL/S: 94.8 % optimal
Alternative 12	EVLT	Equal	Total costs, EVLT	6 weeks	EVLT: 54.9 % CE
Alternative 14	EVLT	Equal	Total costs, EVLT	12 weeks	EVLT: 83.0 % CE
Alternative 25	EVLT	HL/S	Total costs, EVLT	Day 28	HL/S: 99.9 % optimal

**Table 6:** Incremental cost-effectiveness calculations for the sensitivity analyses with the dominance report: “No strategies were clearly dominated by any other”

Strategy	Cost (€)	Incremental cost (€)	Effectiveness (QALY)	Incremental effectiveness (QALY)	Cost-efectiveness (€/QALY)	Incremental cost-effectiveness (€/QALY)
Alternative 7						
EVLT	2676.89		0.113		23626.54	
HL/S	5756.33	3079.45	0.119	0.006	48291.39	521940.11
Alternative 8						
EVLT	2676.89		0.360		7435.79	
HL/S	5756.33	3079.45	0.367	0.007	15663.49	410592.89
Alternative 10						
HL/S	924.67		0.161		5761.163	
EVLT	1363.55	438.89	0.171	0.010	7978.66	42200.64
Alternative 11						
HL/S	924.67		0.328		2819.97	
EVLT	1363.55	438.89	0.351	0.023	3884.77	18999.42
Alternative 12						
HL/S	3028.17		0.161		18867.08	
EVLT	3332.13	303.97	0.171	0.010	19497.56	29227.56
Alternative 14						
HL/S	3028.17		0.355		8530.05	
EVLT	3332.13	303.97	0.380	0.025	8768.77	12158.67
Alternative 23						
HL/S	924.67		−0.007		−127540.23	
EVLT	1363.55	438.89	−0.003	0.004	−419554.87	109721.67
Alternative 25						
HL/S	4971.33		−0.007		−685701.15	
EVLT	7030.22	2058.89	−0.003	0.004	−2163144.62	514721.67

**Table 7:** Cost effectiveness analysis of alternative analysis 10

Alternative 10 (6 weeks, direct costs)	EVLT	HL/S	Incremental effect	Incremental cost	
Cost	€1363.55	€924.66			
Effect (QALY)	0.171	0.161	0.010	€438.89	
ICER					€42200.64

For the alternative case scenarios (n = 8) 4–6, and 17 (EVLT: TRW: 7.0d vs HL/S: TRW: 7.6d), 16, 18, and 22 (only direct costs), and 24 (EVLT: TRW: 20d vs HL/S: TRW: 14d), the strategy “EVLT” was dominated by the strategy “HL/S”, as well (Table 3). In all these alternative scenarios, direct and/or total costs were higher for EVLT, compared to HL/S, and in all cases, time to resume work (TRW) was shorter for HL/S, compared to EVLT, or was equal for both procedures, respectively. These results were not influenced by the different time points of analysis or by the various QoL-values.

Regarding the alternative analyses (n = 5) 9, 13, and 15 (EVLT: TRW: 7.0d vs HL/S: TRW: 12.4d), and 19 and 21 (EVLT: TRW: 4d vs HL/S: TRW: 17d), the strategy “HL/S” was dominated by “EVLT” (Table 4). In all these alternative scenarios, total costs were higher for HL/S, compared to EVLT, and in all cases, time to resume work (TRW) was shorter for EVLT, compared to HL/S. Furthermore, QoL-values were better or equal for EVLT compared to HL/S in all these alternative calculations. Again, these results were not influenced by the time point of investigation.

Finally, in eight alternative scenarios (7, and 8 (EVLT: TRW: 7.0d vs HL/S: TRW: 12.4d), 10, 11, and 23 (only direct costs), and 12, 14 (EVLT: TRW: 7.0d vs HL/S: TRW: 7.6d), and 25 (EVLT: TRW: 20d vs HL/S: TRW: 14d)), no strategies were clearly dominated by any other (Tables 5, 7). In six of the eight alternative analyses, EVLT was characterized by better QoL-data and higher direct and/or total costs, compared to HL/S. Time to resume work was only in one alternative analysis (25) shorter for HL/S, compared to EVLT. According to the alternative scenarios 7 and 8, the incremental cost of HL/S was €3079.45, and the incremental effect was between 0.006 QALY and 0.007 QALY. This represented an incremental cost-effectiveness ratio (ICER) for HL/S of € 521940.11/QALY and €410592.89/QALY, respectively. HL/S was more costly and effective, but its ICER was greater than the WTP, so EVLT was optimal with a certainty of 83.9 % in alternative analysis 7, and a certainty of 89.9 % in alternative analysis 8, respectively. In the alternative analyses 10, 11, 12, 14, 23, and 25, the incremental cost-effectiveness ratio (ICER) for EVLT for uncomplicated varicose veins and evidence of saphenofemoral reflux was positive at between €12158.67 and €514721.67 (Table 7).

Under these conditions, EVLT was only cost-effective at € 50,000/QALY, with a certainty of 58.9 % in alternative analysis 10, 98.8 % in alternative analysis 11 (Fig. 4a, b), in 54.9 % in alternative analysis 12 (Fig. 5a), and in 83.0 % in alternative analysis 14. EVLT was more costly and effective, but its ICER was greater than the WTP, so that HL/S was optimal with a certainty of 94.8 % in alternative analysis 23, and with a certainty of 99.9 % in alternative analysis 25, respectively.

## Discussion

The standard surgical treatment of the insufficient great saphenous vein, high ligation and stripping, and the alternative teatment option, EVLT, are currently an established part of clinical practice. However, the cost-effectiveness of these therapeutic strategies has not been fully investigated in the past. The fact that these procedures are well accepted and widely used creates some difficulty in performing new research work in this area. In recognition of these potential difficulties, the present study was intended to analyse the cost-effectiveness of EVLT and high ligation/striping for varicose veins alongside the results of RCTs by using a range of approaches, including systematic literature review, and economic analysis and modelling.

The economic analysis regarding direct costs showed that over a 6 month period there was an additional cost associated with EVLT of €466.66 with a measured disutility of between 0.0 and −0.070 QALY, so that EVLT was dominated by HL/S in the base-case analyses. By using these calculated cost and utility estimates, we suggest that EVLT is a cost-effective alternative to HL/S with a certainty of only 12.49 % in our base case 1, and of 0.46 % in our base case 2, at a threshold of € 50,000/QALY.

For the alternative case scenarios (n = 8) 4–6, 16, 17, 18, 22, and 24, the strategy “EVLT” was dominated by the strategy “HL/S”, as well. This was caused by the estimates for the costs, which were higher for EVLT compared to HL/S in any of these alternative scenarios, and the estimates for the time to resume work, which were equal between EVLT and HL/S or were shorter for HL/S compared to EVLT.

Regarding the alternative analyses 9, 13, 15, 19, and 21 (n = 5), the strategy “HL/S” was dominated by “EVLT”. In all these scenarios, surgery was characterized by a prolonged time to resume work, namely 12.4 and 17 days, respectively, compared to EVLT with 7.0 and 4.0 days, respectively, and by higher total costs compared to EVLT.

The results of the alternative economic evaluation 10, 11, 12, and 14 indicated EVLT to be a potentially attractive, cost effective (i.e. incremental cost effectiveness ratio of between €12158.67 and €514721.67 per QALY, respectively) treatment option compared to conventional surgical treatment for varicose veins with a certainty between 54.9 and 98.8 %. Thus, in the scenarios 12 and 14, EVLT was associated with comparable times to resume work but with higher total costs and better QoL-data, compared to HL/S. Nevertheless, like in the other alternative scenarios (7, 8, 10, 11, 12, 14, 23, 25), no strategies were clearly dominated by any other.

As a result, in the majority of cases, where conventional surgery was characterized by shorter, similar or only slightly prolonged time to resume work compared to EVLT, and by higher costs compared to EVLT, calculation of costs and utilities exhibited conventional surgery to be optimal with a certainty between 84.8 and 99.9 % or to be more cost effective than EVLT.

The economic component of the present study indicated that, for patients with varicose veins and evidence of saphenofemoral or saphenopopliteal reflux, EVLT offers only a modest health benefit for relatively much additional cost with respect to conservative treatment. These conclusions hold, regardless of the score used to calculate QALYs, and using a number of alternative assumptions relating to unit costs for the treatment options. However, as EVLT becomes more widely adopted, it is possible that the costs associated with the equipment will be reduced, increasing the cost-effectiveness of EVLT.

Regarding the costs, several factors could account for the difference between EVLT and HL/S, namely the length of the procedure, the length of the hospital stay postoperatively, the additional cost of equipment used during EVLT, and possibly the cost of treating the complications, and the cost of reinterventions. Because there were no data available on the costs associated with the complications of EVLT, these costs were not formally included in our analysis, although they are, in part, accounted for in the length of hospital stay and the time to resume work.

Several measures of outcome have the potential to be used for the generation of the utilities for cost-effectiveness analysis. In the present study, both SF-36- and VAS-generated societal utilities were applied from various sources. A robust methodology to estimate utility from the SF-36 score has recently become available with the multivariate regression model developed by Nichol [28] that translates SF-36 scores into HUI2-II scores. The Mark II Health Utility Index (HUI2) measures 7 attributes of health status (sensation, mobility, emotion, ognition, self-care, pain, and fertility) [28]. This utility score is anchored by “perfect health” as the highest possible health state and “dead” as the lowest possible health state. Although the preferred method would be to derive utilities from a community sample directly, this prediction equation by Nichol is a validated tool to obtain an estimate of summary utility scores from secondary health status data using the SF-36. VASs give a quick and simple measure of overall HRQoL, and showed changes that were broadly similar to those seen with the SF-6D and EQ-5D [2]. The methods that conform best to expected utility from VAS are the standard gamble (SG) and time trade-off (TTO) instruments [29, 30]. Although both of these have theoretical advantages, they are based on fairly complex interview techniques, which require that the condition in question is evaluated in respect to a risk of death or change in life expectancy. As a result, there might be problems with using such techniques for conditions which have a relatively minor impact on HRQoL, like in varicose veins. In the present study, the standard gamble instrument was used as an alternative parameter. Finally, although the regression method by Kovacs (32) was collected for a different population group with low back pain, for the purposes of the present analysis it was assumed that the relationship held for varicose vein patients.

In general, since EVLT is costly compared with conventional treatment, and the expected benefits are small, it would need a very large clinical trial to demonstrate its cost-effectiveness when compared with conventional surgery. In view of these considerations, it may be difficult to achieve such a trial, and therefore it might be helpful to examine such techniques, in the first instance, through the collection of observational data from various studies and performing economic modelling, like in the present study.

### Study limitations

Although the effectiveness data were of a high quality, being from prospective randomized controlled trials, and the cost data were current and relevant to our analytic perspective, the results of this analysis are intended to be indicative rather than definitive and need to be interpreted with considerable caution. However, where possible and appropriate, data were also verified and recalculated (e.g., to accommodate intention-to-treat analysis).

This economic analysis was based on short-term data (up to 6 months) owing to the lack of adequate follow-up data beyond that time. In practice, one would expect the benefits of surgical treatment to endure over a longer period. The consideration of benefits beyond 6 months would be likely to result in a reduction in the value of the ICER, so further enhancing the cost-effectiveness of surgery. Our analysis did not take into account the costs associated with the treatment of the long term complications and recurrence rates of EVLT, because these data do not exist until now. This might have further biased our analysis in favour of conventional vein surgery. For example, it would take only relatively small increases in morbidity (e.g. DVT or PE) with EVLT to reduce the acceptability, safety and, therefore, the potential cost effectiveness of EVLT, as an alternative to conventional surgical approaches. In addition, adjunct or concomitant therapies may have been used in the trials. However, there was no valid way to determine what effect these treatments may have had on general outcomes, if any.

There is considerable debate regarding the most appropriate measures of outcome for cost-effectiveness analysis. In the case of varicose veins, the condition causes little disability, with most patients seeking treatment for cosmetic concerns or relatively minor symptoms affecting the legs. The cosmetic appearance of the leg may be an important factor to many patients. This may not be captured in the measurement of QALYs through generic measures of health status which focus upon factors relating to physical, social and emotional functioning rather than cosmetic appearance per se. Because data were not available on HRQoL after EVLT, we synthesized utility values from SF-36 and VAS data by using different transformation equations [28].

The 1-week absence from work and normal activity reported by Rasmussen [17] for the EVLT and HL/S group is low compared with other studies [2, 22, 23], where almost 2 to 3 weeks absence is reported for surgery but also for EVLT. On the other hand, 1 week for patients treated with EVLT may be a long period, although only few studies dealing with the issue have been published so far. Thus, in one small study, no absence from work after EVLT treatment was reported [17]. However, the time to resume work probably depends on the type of work, and the social security system.

## Conclusions

The results of our study, despite its limitations, represent no clear evidence for the cost-effectiveness of EVLT compared with conventional surgery of the great saphenous vein for primary varicosis.

In those patients for whom both treatments would be considered appropriate, surgery is expected to produce greater average benefit at a lower cost per QALY, making it the preferred option from both the patient and the health service perspective.

Therefore, future research needs to focus in providing unbiased estimates of the relative long-term effects of EVLT in comparison to conventional surgical approaches for varicose veins, especially on evaluating the effect of EVLT on the patient’s quality of life.

## Abbreviations

EQ-5D: EuroQol 5-dimension measure of health-related quality of life
HRQoL: Health-related quality of life
ICER: Incremental cost-effectiveness ratio
NHS: National Health Service
QALY: Quality-adjusted life year
VAS: Visual analog scale
SG: Standard gamble
HUI2-II utility scores: Mark II health utility index
EVLT: Endovenous laser ablation
HL/S: High ligation and stripping
